# The Future of Alopecia Treatment: Plant Extracts, Nanocarriers, and 3D Bioprinting in Focus

**DOI:** 10.3390/pharmaceutics17050584

**Published:** 2025-04-29

**Authors:** Rana E. Elnady, Manar S. Abdon, Hagar R. Shaheen, Reem M. Eladawy, Yasmena O. Azar, Seham M. Al Raish

**Affiliations:** 1 Department of Pharmaceutics, Faculty of Pharmacy, Sinai University—Arish Branch, Arish 45511, Egypt; rana.essam@su.edu.eg (R.E.E.); manar.hassan@su.edu.eg (M.S.A.); hager.rabea@su.edu.eg (H.R.S.); 2 Department of Pharmacology and Toxicology, Faculty of Pharmacy, Sinai University—Arish Branch, Arish 45511, Egypt; reem.ali@su.edu.eg (R.M.E.); yasmena.osama@su.edu.eg (Y.O.A.); 3 Department of Biology, College of Science, United Arab Emirates University, Al Ain 15551, United Arab Emirates

**Keywords:** nanocarriers, phytomedicines, alopecia, nanomaterials, follicle, 3D bioprinting

## Abstract

Alopecia is a concerning dermatological issue and is also known as alopecia. This disease can affect men and women, influencing their confidence and appearance. It targets the scalp or any area of the entire body. Alopecia has become widespread worldwide over the years and has many types and different causes: hereditary, hormonal, immunological, therapeutic, or psychological. This review will present a comprehensive study of the physiological structure of hair and the different growth and shedding phases. It discusses using nano-drug delivery systems that contain natural substances of plant origin, which are effective, less harmful compared to current treatments, and help avoid adverse effects. This review also covers the latest trends in treating alopecia, including drug delivery systems, the materials and methods used to prepare these systems, three-dimensional (3D) bioprinting strategies, and plant extracts that may be utilized for treatment in the coming years.

## 1. Introduction

Hair follicles (HFs) play an essential role in human skin, offering protection, sensation, and thermal regulation [1]. The normal hair cycle includes three essential phases (anagen, catagen, and telogen) [2]. In some cases, the hair cycle phases can be affected by unfavorable triggers leading to hair loss (alopecia). So, alopecia occurs due to immunological factors called alopecia areata (AA) [3], which could further lead to total scalp hair loss, called alopecia totalis (AT), or whole body hair loss, called alopecia universalis (AU) [4]. In some cases, it occurs symmetrically, defined as alopecia ophiasis (AO), or from hormonal factors, called androgenic alopecia (AGA) [5]. Moreover, chemotherapy and some medicines can cause anagen effluvium, a type of alopecia. Stress is also one of the important causes of alopecia, called telogen effluvium [6]. This review focuses on AGA and AA, as they are considered the most important types of alopecia, as other types are reversible and improve by stopping the external causes.

Hair loss caused by AGA generally begins after puberty and is more common in males than females [7]. The prevalence of AGA varies by gender and race, as it affects about 50% of older men and 15% of postmenopausal women [8]. The complex interaction of genetic, hormonal, and environmental variables underlies the pathophysiology of AGA [9]. Genomics research offers compelling proof of the underlying processes of specific genes and their regulation of HF growth and development pathways, emphasizing that altering HF signaling pathways dictates the occurrence of AGA [10]. The 5α-reductase and androgen receptor (AR) genes are attractive candidates [11]. Comparing non-balding follicles with blading follicles on the same scalp, balding follicles have higher levels of expression for the AR and 5-alpha reductase enzymes [12]. Several studies have related high levels of dihydrotestosterone (DHT) and the overexpression of the androgen receptor gene to the pathogenesis of AGA. The 5α-reductase (5-AR) enzyme, which is found in the dermal papilla, converts testosterone from the blood circulation and HFs into DHT [13]. DHT binds to the androgen receptor within the HF, activating signaling pathways that produce shrinkage and hair loss [13].

In addition, AA is an autoimmune disease characterized by inflammatory T cells attacking the HF region [14]. According to the statistics, AA is a widespread disorder that affects individuals worldwide and usually occurs in a clinical environment [15]. The disease often manifests as round or spotty bald areas with no scars [3].

## 2. Hair Structure and Cycle

**Figure 1:**
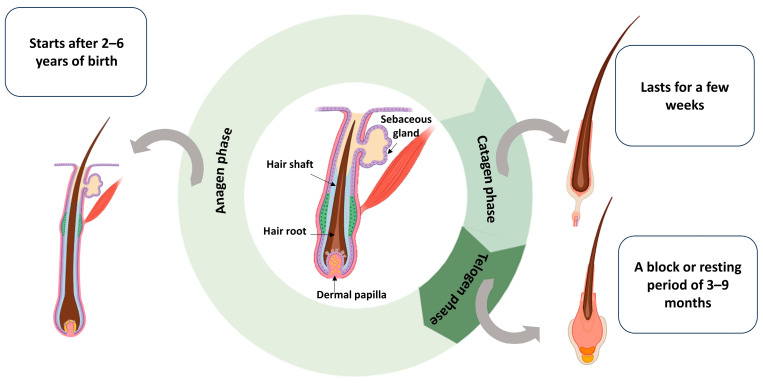
Hair structure and cycle throughout different stages.

Hair and plants are very similar in taking nutrients from their roots. The hair is supplied with growth factors through the HF, which is supplied with blood vessels that contain various growth factors to begin forming the different hair stages. If there is an imbalance in these factors, it will cause hair loss and diseases. Hair fiber originates from a region called the HF; it consists of three zones: protein and cell synthesis, differentiation, and keratinization. The protein and cell synthesis zone is the structure area and contains the papillae responsible for hair growth in the anagen phase [16]. Anagen is a growth phase that starts after 2–6 years of birth, followed by catagen, a transition phase that lasts for a few weeks in which the hair shaft starts to migrate to the skin surface to be renewed again after a block or resting period of 3–9 months, called the telogen phase, until another anagen is formed [16,17]. Hair forms through the rapid division and differentiation of stem cells, forming keratinocytes that migrate, flatten, and die, forming keratinized cells, with the cyclical growth of the HF [18]. Hair keratins represent up to 95% of the hair structure and are produced by special hair follicular keratinocytes that are activated inside the hair bulb by mediators released by dermal follicular cells [19]. Figure 1 represents the structure and cycle of the HF.

## 3. Current Strategies in Alopecia Treatment

### 3.1. Non-Therapeutic Treatment

#### 3.1.1. Hair Transplantation

Autologous hair transplantation surgery involves moving healthy hair to bald or thinning parts of the scalp, taking several hours under anesthesia. The results of hair transplantation are permanent and cost-effective; however, they may involve many side effects due to anesthesia, scarring formation, bleeding, edema, intraoperative or postoperative pain, and patient discomfort [20]. Side effects depend on the technique of transplantation used.

Follicular unit extraction is a surgical technique in which the HF is taken from the donor area (back of the head) with special micropunches and transferred to the affected area [21].

The follicular unit transfer is performed by cutting a thin strip of scalp from the back of the head, and the HF is extracted and transplanted into the recipient area; that technique may leave a small, linear scar on the donor site [22]. Androgenic alopecia has a high success rate of over 90% with this technique; however, for alopecia areata it has a limited impact due to immunological attacks on the transplanted hair [21].

#### 3.1.2. Platelet-Rich Plasma (PRP)

The plasma treatment promotes and maintains hair growth by taking a blood sample from the patient’s vein, separating plasma from red blood cells using centrifugation, and then injecting it deeply into the dermis or subcutaneous tissue [23]. PRP induces minor side effects, like pain, a burning sensation, and a headache that lasts from 10 to 15 min post-injection; moreover, PRP should be avoided in patients with bleeding disorders, autoimmune diseases, active infections, or anticoagulant medications [24].

#### 3.1.3. Stem Cells

This therapy promotes HF growth and regeneration by reactivating HF stem cells. There are two types of transplants: autologous and allogeneic [25]. Stem cell transplantation poses risks such as tumor formation, stem cell inappropriate migration, immune rejection, hemorrhage during surgery, and postoperative infection [26].

#### 3.1.4. Exosomes

Recent studies revealed that exosomes derived from mesenchymal stem cells significantly impact hair growth and generation due to their ability for secretion and multi-directional differentiation [27,28]. Moreover, loading herbal Chinese extracts into exosomes was discovered to be a promising candidate for hair proliferation [29]. Exosomes can withstand the challenges associated with synthetic materials, like recognition by the immune system, while entering the human body [30]. Although the potential ability of exosomes in hair restoration is excellent, more controls are needed for the subsequent exosomal modifications, which restrict their use as they must be isolated and purified with strict quality, purity, potency, and repeatability requirements [31].

#### 3.1.5. Low-Level Light Therapy (LLLT)

The technique of LLLT is safe, non-invasive, and convenient for hair regrowth and density, with a 95% success rate and results visible within 4 months [32]. LLLT devices, like Capillus^®^ and Hairmax^®^, use red light absorption to stimulate mitochondrial cytochrome c oxidase, causing the higher production of ATP, reactive oxygen species, and the transcription factor [33]. Like laser therapy, LLLT could cause some complications, such as burns, infections, dyspigmentation, ophthalmic injuries, scarring, prolonged erythema, acne, and contact dermatitis [34].

#### 3.1.6. Microneedling

Microneedling (MN) is a safe and effective therapy that stimulates hair regeneration by releasing growth factors and increasing the density and thickness over 12 weeks using 0.6 mm needles [35]. Although MN enhances the absorption of medications, it leaves the skin red and inflamed. Another type is called candlelit-dissolving MN, which dissolves after the penetration and transportation of drugs [36].

### 3.2. Therapeutic Treatment

Many strategies in alopecia therapy have been conducted. Due to the diversity of alopecia types, treatment protocols cannot be fixed for all patients.

#### 3.2.1. Topical Corticosteroids (TCs)

Corticosteroids are the most common treatment for AA for small patches in adults and children. Corticosteroids reduce inflammation, suppress the immune system around HFs, and increase hair regeneration [37]. The treatment must be continued for at least 3 months and revealed a 70% hair regrowth rate, and maintenance therapy is required [38]. Topical corticosteroids are available in various formulations, including creams, lotions, gels, mousses, ointments, tapes, bandages, and solutions [39]. Stacey et al. (2021) found that topical corticosteroids (high potency group I and II) are effectively treat AA [39].

#### 3.2.2. Intralesional Corticosteroids

They are the first-line treatment for localized conditions involving <50% of the scalp [40]. Hydrocortisone acetate injections (25 mg/mL) and triamcinolone acetonide (5–10 mg/mL) are commonly used. Triamcinolone acetonide is injected locally every 4–6 weeks and reaches 95% of hair regrowth at 24 weeks [41]. Skin atrophy is a common side effect at the injection site when triamcinolone is used [42]. Studies report hair regrowth in up to 64% of treated areas for localized alopecia areata in adults [43].

#### 3.2.3. Systemic Corticosteroids

The suggested doses for systemic corticosteroids in adults with severe or rapidly progressive AA ranged from 0.5 to 1 mg/kg per day, while children should take 0.1 to 1 mg/kg daily [44,45]. Systemic corticosteroids are a more limited alternative due to their side effect profile, long-term treatment requirements, and significant recurrence rates [41]. A study by Kirchner et al. (2000) reported that oral corticosteroids therapy may be effective, but probable adverse effects usually prevent its use [46].

#### 3.2.4. Contact Immunotherapy

Topical immunotherapy is currently the best-documented treatment for severe and refractory AA. The contact allergens utilized for this purpose are dinitrochlorobenzene (DNCB), squaric acid dibutylester (SADBE), and diphencyprone (DPCP) [47]. SADBE suffers from many side effects, including persistent dermatitis, painful cervical lymphadenopathy, generalized eczema, blistering, contact leukoderma, and urticarial reactions; moreover, systemic manifestations include fever, arthralgia, and yellowish discoloration [48]. Moreover, a study demonstrated that the overall regrowth rate of contact immunotherapy was 65.5% among AA patients; however, complete regrowth was observed in only 32.3% of them [49].

#### 3.2.5. Topical Minoxidil

The topical minoxidil solution (1% and 5%) is an adjunct therapy and has better results in mild cases; it is a direct-acting arteriolar vasodilator, which is explicitly used to open the potassium channels to increase the amount of intracellular Ca^2+^, which in turn upregulates the enzyme adenosine triphosphate (ATP) synthase to promote stem cell differentiation that plays a key role in the facilitation of hair growth [50,51]. An El Taieb et al. study found that minoxidil is effective in 81% of cases with patchy AA [41]. Minoxidil induces side effects like headaches, allergic and irritant contact dermatitis, and hypertrichosis [52]. The increase in minoxidil’s adverse reactions leads to treatment discontinuity and hampers patients’ compliance with the treatment, limiting its therapeutic success [53]. Moreover, the low solubility of minoxidil induces a challenge in producing a non-irritant and effective formulation [54].

#### 3.2.6. Finasteride

Since 1997, oral finasteride has been authorized for the management of male pattern baldness (AGA). The medication works by preventing the conversion of testosterone to DHT by inhibiting the Type II 5 alpha reductase enzyme [55]. A well-known, extensive Japanese study including over 3000 male participants with AGA showed that, after using finasteride for three years, 11.1% of subjects had significant hair regrowth, 36.5% showed moderate growth, and 39.5% only showed a slight rise in hair growth [56]. Finasteride as a topical formulation is available from compounding pharmacies, and at least some formulations have been shown to significantly reduce plasma and scalp DHT levels [57]. Compared to the oral form, the topical finasteride gel has demonstrated a similar efficacy [58]. Side effects from oral finasteride include orthostatic hypotension and sexual side effects [54]; moreover, the data linking the use of finasteride to reproductive problems are conflicting, and patients who are considering finasteride treatment frequently voice worries about the possibility of infertility while on the medication [21]. Moreover, topical finasteride includes skin erythema, contact dermatitis, increased liver enzymes, nocturnal enuresis, testicular pain, headaches, presyncope, and oropharyngeal pain [57].

#### 3.2.7. Dutasteride

Dutasteride functions as a selective competitive inhibitor of type 1 and type 2 isoenzymes of 5-alpha-reductase and a second-generation inhibitor of 5-alpha-reductase [59]. It has been observed that dutasteride is 100 times more effective than finasteride in inhibiting type II enzymes and three times more potent at inhibiting type I enzymes [60]. Compared to finasteride, dutasteride has demonstrated a greater efficacy in preventing DHT and encouraging hair growth. Dutasteride was shown to block 98.4% of DHT in a trial including 399 participants, whereas finasteride blocked roughly 70% [60]. Another study that included 416 men between the ages of 21 and 45 discovered that dutasteride outperformed finasteride over 12 to 24 weeks in terms of hair count results [27]. Despite the better efficacy shown by dutasteride, finasteride is still likely to be recommended more frequently as a first-line medication in treating AGA due to its Food and Drug Administration (FDA) approval and insurance coverage [21]. Therefore, an effective, localized dutasteride treatment that can reduce the effects of systemic uptake is of great interest [61]. The adverse effects of oral dutasteride are comparable to those of finasteride and include a decreased libido, erectile dysfunction, and ejaculatory dysfunction [62].

#### 3.2.8. Sodium Valproate

A popular medication used by many different disciplines is valproate. Upon oral administration it is linked to hair loss, which is a cosmetic issue. However, preliminary findings suggest that topical valproate treatments encourage hair growth [63]. Topical valproate was tested in a double-blind, randomized, placebo-controlled clinical trial to determine its effectiveness in treating androgenic alopecia. For a duration of 24 weeks, male patients diagnosed with mild androgenic alopecia were treated with either a placebo spray or VPA (sodium valproate, 8.3%). The change in the hair count during therapy, measured by phototrichogram analysis, was the primary endpoint for effectiveness. A progressive dose increase is crucial in counteracting valproate-induced hair loss, weight gain, tremors, liver dysfunction, gastrointestinal disturbances, thrombocytopenia, and metabolic acidosis [64]. Compared to the placebo group, the VPA group experienced a considerably more significant mean change in total hair count [65]. However, more research is needed [43].

### 3.3. Phytochemical Treatment

There are currently several synthetic therapies available to treat hair loss (areata and androgenetic), including dithranol, corticosteroids, minoxidil, tretinoin, minoxidil, zinc, irritants, immunosuppressants, azelaic acid, systemic cortisone, and finasteride. However, none of these therapies have been demonstrated to have beneficial and long-lasting effects on patients. These drugs are also associated with synthetic adverse effects, such as scaliness, erythema, pruritus, itching, dermatitis, etc. Therefore, we looked to nature’s treasures to solve the hair loss problem. Several herbs have been proven to be effective in treating hair loss by several mechanisms, such as increasing the scalp blood circulation, DHT, 5α-reductase blockers, aromatherapy, and nutritional support. Herbal remedies for alopecia include *Phyllanthus emblica*, *Oscimum sanctum*, *Aloe barbadensis*, *Allium cepa*, *Allium sativum*, *Eclipta alba*, *Thea sinensis*, *Cocas nucifera*, *Centella asiatica*, *Trigonella foenum graecum*, and *Sesamum indicum* [66].

#### 3.3.1. Mechanism of Phytochemicals in Alopecia

##### Aloe Vera (*Aloe barbadensis*)

Aloe vera includes vitamins A, C, and E, strengthening and repairing hair strands [67]. These three vitamins encourage healthy cell development, support cell regeneration, and add shine to hair. Aloe vera gel also contains folic acid and vitamin B12 so that it can prevent hair loss [67]. Vitamin B12 and folic acid promote nucleic acid, which is essential in the formation and replication of cells and HF growth [68]. In aloe vera, aloenin is the main constituent promoting hair growth in alopecia [67]. Aloenin can stimulate the growth of human dermal papilla cells through the Wnt/β-catenin signaling pathway activation, which is required for HF development and cycling. These cells are essential for transitioning the HF into the anagen phase [69]. Studies suggest aloe vera may improve hair growth in AGA and AA [70].

Additionally, some people with mild to moderate alopecia reported increased hair growth after applying topical aloe vera. A topical gel is applied directly to the scalp; shampoos and conditioners are frequently mixed with other substances that promote hair growth, such as saw palmetto and minoxidil. Furthermore, some research indicates that aloe vera used internally, for example, as juice or as an oral supplement, may promote hair health. A previous clinical study found that aloe vera gel, along with minoxidil, enhanced hair growth in AGA [71], and there are approved shampoos and serums containing aloe vera on the market. Aloe vera has shown promise in treating some forms of alopecia, but more extensive clinical studies are required to verify its effectiveness compared to more traditional treatments like finasteride or minoxidil. Current research supports its use as a supportive therapy for minor hair loss and scalp health [72].

##### Amla (*Phyllanthus emblica*)

The antimicrobial properties of amla help prevent dandruff and other fungal infections and improve the health of the scalp [73]. Amla and finasteride, used to treat hair loss in both men and women, are considered potent inhibitors of 5α-reductase [73]. Amla treats hair loss in both men and women, prevents dandruff, improves the scalp’s health, and purifies the blood supply [73]. Amla (*Phyllanthus emblica*) shows promise as an effective and safe treatment for female AGA. Its oral syrup form improves hair growth parameters and satisfaction levels among patients. Further comparative studies with standard treatments like finasteride are needed to establish its relative efficacy [74]. A recent randomized controlled clinical trial was conducted on the effect of an oral product containing the amla fruit on AGA [74], and there are approved oil products with the amla herb.

##### Onion (*Allium cepa*)

The zinc content in onions helps produce essential oils for the scalp and prevents the hair loss associated with dandruff [67,73]. In addition to iron’s essential role helping RBC carry oxygen, it helps develop healthy hair. The sulfur content in onion is good for strengthening or thickening hair [67,73]. Onion prevents hair loss, promotes hair growth, strengthens or thickens hair, and produces healthy skin cells and hair growth, which is supported by collagen [67,73]. Onion juice is an effective topical treatment for AA, with high success rates observed in clinical trials. Male participants responded better (93.7%) than females (71.4%), highlighting onion juice’s effectiveness as a topical treatment for AA [75]. Its simple application method and minimal side effects make it a promising alternative or complementary therapy for individuals experiencing patchy hair loss. A previous clinical study on onion juice for the treatment of AA comparing onion juice with conventional treatments, like minoxidil, could provide deeper insights into its therapeutic potential [75].

##### Garlic (*Allium sativam*)

Garlic has antibacterial properties, which kill germs and bacteria that cause damage to the scalp and hinder hair growth [76]. The primary bioactive chemical found in garlic is generated when it is enzymatically transformed by alliinase while crushed or chopped [77]. It is known that garlic has a high vitamin C content, which is good for hair health [78]. It also stimulates collagen production, which contributes to hair growth. Garlic is used to promote hair health and growth [76]. Garlic oil can control bacterial attacks on the scalp in AA [79]. Garlic is a cost-effective and potent natural remedy for AA. Its topical application in a gel or oil form demonstrates significant success rates in clinical studies. With its antioxidant, antimicrobial, and anti-inflammatory properties, garlic addresses multiple factors contributing to hair loss. Further research comparing garlic treatments with conventional therapies could solidify its role in dermatology [80,81]. A previous clinical study on garlic was implemented using a combination of topical garlic gel and betamethasone valerate cream to treat localized AA [82]. Additionally, there are approved cream products containing garlic on the market.

##### Bhringraj (*Eclipta Alba*)

*Eclipta Alba* methanol extract promotes HF anagen during the telogen phase and increases hair growth [67,73]. *Eclipta Alba*’s vitamin E content nourishes the scalp, strengthens the hair strands, and moisturizes the skin, making it luminous and healthy. Eclipta Alba nourishes the scalp and HF, supporting the HF in regrowing more hair [67,73]. *Eclipta Alba* is highly successful in promoting hair regrowth, with topical application of its extracts or oil being the standard and effective form. It is indicated for various types of alopecia, including AGA and chemotherapy-induced alopecia management, through mechanisms involving follicular stimulation, anagen phase extension, and scalp health improvement. These findings are supported by experimental studies and traditional Ayurvedic use, warranting further clinical trials to confirm its efficacy in humans [41,83]. Many experimental studies on *Eclipta Alba* are available for hair growth [84,85]. However, no definitive clinical studies have proven its hair growth efficacy.

##### Tea (*Thea sinesis*)

The caffeine in tea stimulates HFs and leads to an increased scalp blood flow [78,86]. Epigallocatechin gallate (EGCG) in tea inhibits hair loss by preventing the hormone activity that triggers hair loss and enhances hair growth by HF stimulation [78,86,87,88]. A recent study proved that a polyphenol complex containing different ratios of tannic acid, gallic acid, and caffeic acid enhanced the self-recovery of damaged hair while protecting the cuticle [89]. Tea, particularly its active compound EGCG, shows promise as a natural remedy for alopecia due to its ability to stimulate dermal papilla cells, inhibit DHT production, and reduce oxidative stress. The topical application of green tea extracts or EGCG-enriched solutions appears effective in preclinical studies, but further clinical trials are needed to confirm its efficacy in humans. Its dual antioxidant and anti-inflammatory actions make it a valuable adjunct therapy for managing various forms of alopecia, such as AA, AGA, and oxidative stress-induced hair loss [90,91]. Additionally, a former study evaluated a green tea hair tonic for greasy scalp treatment [92].

##### Fenugreek (*Trigonella foenum graecum*)

Fenugreek seeds contain proteins, carbohydrates, lipids, flavonoids, alkaloids, fibers, saponins, steroidal saponins, minerals, vitamins, and nitrogen compounds classified as non-volatile or volatile [93]. Several substances in fenugreek may react with a substance in the human body called dihydrotestosterone (DHT). DHT binds to HFs and eventually causes hair loss. Fenugreek may decrease DHT’s capability to attach to the HF [93]. Fenugreek is high in proteins and amino acids, which help to repair hair shafts that heat styling, dehydration, compounds, or sun exposure have impaired. Fenugreek increases the cuticle’s integration into the hair shaft and substantially supports hair growth [93].

Additionally, an animal study revealed that ethanol extracts of fenugreek cause significantly improved hair length and growth compared to 5% minoxidil, a standard treatment for alopecia (*p* < 0.05) [94]. Fenugreek is indicated for various types of alopecia, including AA, AGA, and hormonal hair loss [95]. Topical applications, such as masks, rinses, and oils, have shown significant efficacy in preclinical studies. While further human trials are needed to confirm its effectiveness, fenugreek’s multifaceted benefits make it a viable adjunct therapy for managing alopecia. Interestingly, fenugreek seeds can be consumed as part of the diet or as supplements to provide systemic benefits, including hormone regulation, that may indirectly support hair health [96]. A randomized, placebo-controlled clinical trial was conducted to evaluate the efficacy of a fenugreek-seed-containing food supplement against hair loss. The results showed a successful treatment of low to moderate hair loss in women and men [97].

##### Coconut (*Cocos nucifera* L.)

Coconut oil contains lauric acid, attaches to hair proteins, and protects the roots and strands from injury [93]. Coconut oil’s antioxidants promote strong hair growth [93]. Coconut oil is known for penetrating the hair shaft and protecting it from environmental contaminants and excessive heat [93]. Coconut oil’s anti-fungal and antibacterial properties help to protect against bacterial infections that can hamper hair growth [93]. Coconut is used in hair hydration to reduce damage [93]. A clinical study evaluating a hair serum containing freeze-dried coconut water alongside other active ingredients reported significant improvements in hair growth parameters after 90 days of daily application. The study showed a statistically significant increase in the hair growth rate (17.36% improvement), hair density, and the density of both vellus and terminal hairs (all *p* < 0.0001) [98]. Coconut exhibits a high success rate in improving hair growth and reducing hair fall when applied topically in formulations containing coconut water or oil. Its rich nutrient profile supports the HF’s health and scalp condition, making it a safe and effective adjunct for AGA, hair fall reduction, and general hair thinning. Coconut is used in hair hydration to reduce damage [93]. Further large-scale clinical trials could consolidate its role in dermatological practice [99]. Clinical research on the formulation and establishment of the efficacy of coconut oil was employed and showed a successful treatment in hair loss [100].

##### Almond (*Prunus amygdalus*)

Almond oil contains biotin, so rubbing it into the scalp is a good way to provide adequate vitamins to promote hair development and prevent its thinning [67,93]. Rubbing almond oil on the epidermis and scalp to increase blood flow through the hair roots results in hair development and strength [67]. It helps to renew and generate sufficient keratin to make hair thicker [67]. Almond offers promising benefits in managing alopecia through the topical application of its oil or seed meal formulations. While clinical data on its specific success rate for alopecia remain limited, its safety profile and traditional use support its effectiveness as a natural remedy for improving scalp health, reducing hair thinning, and promoting regrowth. Further clinical studies are needed to quantify its efficacy compared to conventional treatments [101].

##### Tulsi (*Oscimum sanctum*)

Tulsi has a beneficial impact on the hair, revitalizing the HF and reinforcing the hair roots to prevent hair loss [67]. This herbal treatment boosts blood flow through the scalp and keeps it cool [67]. Tulsi has been utilized to avoid hair loss and thinning while improving hair thickness [67]. Tulsi’s anti-inflammatory ingredients may irritate the scalp [67]. Although high-quality clinical trials specifically quantifying Tulsi’s success rate in alopecia are limited, the preliminary evidence and traditional use suggest positive outcomes. A 2011 study reported an improvement in alopecia symptoms with Tulsi aromatherapy, indicating its potential to stimulate hair growth. Tulsi exhibits multifaceted properties, such as anti-inflammatory, antioxidant, antimicrobial, and adaptogenic properties, collectively supporting its use in AA management. However, further rigorous clinical trials are necessary to establish standardized protocols and quantify their efficacy. Its versatile application forms, including topical, oral, and aromatherapy, make Tulsi a promising adjunct in holistic hair loss treatment strategies [102]. For example, a previous clinical perspective demonstrated the effectiveness and safety of a Tulsi herbal shampoo against alopecia and seborrheic dermatitis [103].

#### 3.3.2. Limitations of Phytochemicals

Phytomedicine, a significant source of medication since antiquity, now accounts for almost half of all effective drugs. Its rising use is attributed to its superior therapeutic effectiveness and fewer adverse effects when compared to allopathic drugs. However, phytomedicines have a poor in vivo efficacy due to their low water solubility, lipophilicity, and molecular size [104]. Creating nanoparticles containing herbal extracts is known as “green technology” since it does not require harmful chemicals. Green techniques primarily use harmless biomolecules, such as carbohydrates, DNA, enzymes, proteins, and plant extracts, to make biocompatible NPs [105]. Recently, nanotechnology has developed delivery methods tailored to improve natural bioactive compounds’ therapeutic efficacy. Indeed, nanocarriers have received increased interest as viable alternatives to classic formulation procedures that reduce toxicity, increase bioavailability, and allow for a site-specific targeted delivery [106].

#### 3.3.3. Safety and Regulatory Concerns of Phytochemicals

While many phytochemicals are mostly considered harmless, some have been accompanied by some adverse effects. Aloe vera is generally safe when applied topically, but its oral consumption is harmful and has been regulated by the FDA for many years [107]. While amla is generally considered safe when used moderately. However, extreme use may cause gastrointestinal problems, such as bloating or diarrhea, due to its high vitamin C content. Amla is typically regulated as a food supplement and must adhere to appropriate dosage guidelines [108]. Onions may cause contact dermatitis [109]. Garlic may cause bleeding risk (antiplatelet effect), GI upset, and allergic reactions [110]. Onion and garlic are generally recognized as safe by the FDA. Bhringraj has limited toxicity data, but it may cause potential hepatotoxicity at high doses [111] and is not FDA-evaluated. Tea has caffeine, which may cause insomnia, hypertension, and liver toxicity (green tea extracts), as well as tannins, which may inhibit iron absorption [112]. Tea is regulated as a food product by the FDA. Fenugreek may cause hypoglycemia, allergic reactions, and uterine stimulation and should be avoided in pregnancy [113]. It is generally recognized as safe in the US, but it is restricted in some EU countries. Coconuts contain high amounts of saturated fats, which may cause heart problems [114]. It is generally recognized as safe by the FDA. Raw bitter almonds contain toxic cyanogenic glycosides [115]. Sweet almonds are approved, while bitter almonds are restricted. Tulsi contains Eugenol, which may cause liver toxicity at high doses [116]. It is regulated as a dietary supplement in the US and as a traditional medicine in Ayurveda.

## 4. Nano-Drug Delivery Systems in Loading Phytochemicals for Alopecia

**Figure 2:**
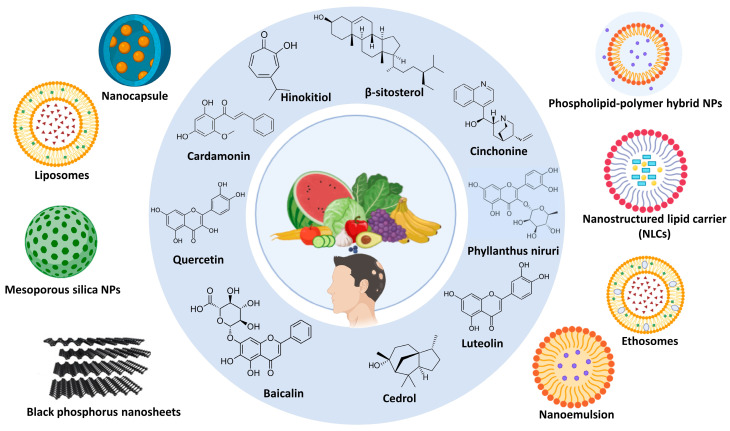
Nano-drug delivery systems loading phytochemicals for alopecia treatment.

Recently, nanosystems demonstrated their potential in treating different types of alopecia. They also have intriguing opportunities for improved HF regeneration therapy in alopecia due to their targeted distribution, high penetration, controlled release, increased bioavailability, and biocompatibility [117]. Figure 2 illustrates different nano-drug delivery systems loading phytochemicals for alopecia treatment.

### 4.1. Niosomes

Niosomes are non-ionic surfactant nanovesicles with innovative drug delivery methods that improve the solubility and stability of natural medicinal compounds, allowing for a targeted and controlled release [118]. Cholesterol can impact the structure of niosome vesicles because hydrogen interactions between hydroxyl groups and the alkyl chains of surfactant molecules increase the bilayer stability [119]. Niosomes were examined as a transdermal drug delivery technology, focusing on improving penetration and reducing skin irritation across the stratum corneum [120]. Niosomes, with their lamellar structures of nonionic surfactants and cholesterols, may aid in the penetration of active components through the skin. Moreover, oil-soluble actives in niosomes have a better chance of permeating and accumulating in HFs than regular oil solutions [121].

### 4.2. Zinc Mesoporous Silica Nanoparticles

Mesoporous silica nanoparticles have been examined as one of the most promising drug delivery systems because of their outstanding biocompatibility and porous structure, which is optimal for drug loading [122]. The necessity of preventing premature medication leakage from the pores in the bloodstream and ensuring they reach the correct cells by targeting is a significant problem [119]. A previous study proved that a systematic intervention utilizing multi-component zinc mesoporous silica nanocomposite biomaterials that simultaneously target three pathophysiological processes is an effective way to treat AGA, which could be used to develop novel therapies for the clinical treatment of AGA and other types of hair loss [123].

### 4.3. Phospholipid–Polymer Hybrid Nanoparticles

They are core–shell nanocarriers that combine polymers and phospholipids in their structure. This combination offers standard features between the nanoparticles and liposomes, enhanced stability, biocompatibility, and cellular uptake [124]. Lipid/polymer hybrid vesicles observed lower diameters than the polymersomes and liposomes, while the encapsulation efficiency and content release performance of the hybrid nanovesicles can be adjusted by mixing different ratios of the amphiphiles [125]. Quercetin-loaded phospholipid hybrid NPs could be included in the treatment of androgenic alopecia, and their antiandrogenic activity potential can be further increased along with novel drug delivery systems, such as dipalmotylphosphatidylcholine (DPPC)-linked poly lactide-co-glycolide (PLGA and DPPC-PLGANPs). Due to their lipophilicity, DPPC-PLGA hybrid NPs can adhere to the scalp effectively [126]. Table 1 describes the in vivo significance of several phytomedicinal-loaded nanoparticles.

**Table 1:** The potential of nanoparticles in loading phytomedicines in treating alopecia.

Phytomedicine	Source from Plant	Nanovesicle	Materials	Method	Animal	In Vivo Outcomes	Ref.
Pumpkin Seed Oil (PSO)	Seeds	Niosomes	Tween 20, cholesterol	Ethanol injection method	Pig ear skin	PSO-loaded niosomes inhibited the mRNA expression of the synthesis genes of 5-reductase. Showed anti-inflammatory activity. Better skin permeation and accumulation of niosomes compared to the PSO solution. Significant decrease in the percentage of fallen hairs by 44.42%. Significant increase in the anagen to telogen (A/T) ratio (1.4-fold).	[121]
Quercetin (Qu)	Skins, peels, outer leaves, and flowers that are found in red onions(skin), capers, berries, kale, and buckwheat	Zinc mesoporous silica	Zinc nitrate hexahydrate, sodium alginate, cetyltrimethylammonium bromide, tetraethyl orthosilicate, copper nitrate trihydrate, ammonium fluoride, and copper nitrate trihydrate	Sol–gel method	C57BL/6 mice	The hair regrowth of the ZCQ/MN group reached 95.33% on the 14th day compared to the blank group. The cytokeratin 19 (CK19) and hematoxylin-eosin (H&E) staining presented that ZC/MN, Qu/MN, and ZCQ/MN stimulated the development and HF growth. Combining Zn/Cu ions with Qu results in the most effective HF growth and maturation stimulation. Combining Zn/Cu ions with Qu enhances HF development and maturation.	[123]
		Phospholipid–polymer Hybrid nanoparticles	Polyvinyl alcohol (PVA), Ethyl acetate, 1, 2-Dipalmitoylsn-glycero-3-phosphocholine (DPPC)	Double emulsification Solvent evaporation	Sprague–Dawley male rats	Hybrid NPs enhanced Qu’s regrowth of hair. NPs accumulation at the HF’s end region inhibited HF cell apoptosis. Qu NPs was compared to minoxidil 5%.	[126]
Cinchonine (CN)	Cinchona bark	NLCs	Stearic acid, oleic acid, polysorbate 80, and glycerin	Combination of microemulsification and ultra-sonification	Swiss Webster male mice	The NLCs delivery system boosts CN activity. It promotes hair growth and regrowth in AA conditions. It promotes the nutrients and oxygen supply required for hair growth and regeneration. It accelerates the anagen phase of HFs and dermal papillae by activating Wnt/β-catenin, (crucial for follicle morphogenesis, development, and growth). It was compared to minoxidil 2%.	[127]
Carthamus tinctorius florets extract	Safflower florets		Monostearin, capric/caprylic triglycerides, Brij-L4, span 60, Tween 60, and Pluronic F-68	Hot high-pressure homogenization	C56BL/6Mlac male mice	The hair growth-promoting activity of the NLCs base was equal to the minoxidil. NLCs increased the hair growth-promoting activity of safflower. NLCs were compared to minoxidil 2%.	[128]
β-vulgaris L. Extract (BVEN)	Roots	Nanoparticles incorporated into a gel	Chitosan, sodium alginate, calcium chloride, acetic acid, sodium hydroxide, carbopol 934, methyl paraben, propyl paraben, and propylene glycol	Ionic gelation	Male Swiss albino mice	Finasteride 2% and 5% BVEN-treated groups showed a higher significant hair growth, while the 2.5% BVEN-treated group showed a less significant hair growth compared to the blank nanogel-treated group.	[129]
β-sitosterol	Seeds, nuts, and oily fruits, or the vegetable oils derived from them	NLCs	Glyceryl mono stearate, virgin coconut oil, and Tween 80	High-speed homogenization	Male Wistar rats	Enhanced permeation and hair growth by loading β-sitosterol using chitosan MN. It was compared with a drug solution.	[130]
Phyllanthus niruri	Root	Ethosomes	Ethanol, Propylene glycol, Soya lecithin	Hot method	Male Wistar rats	The ethosomal formulations containing extracts inhibited testosterone-induced hair loss. The combined ethanol extracts-loaded ethosome formulation produced less cytotoxicity than the combined pet. ether formulation. The high ratio of anagenic HF indicates a restoration of hair loss induced by androgen. There was no observed redness in the combined ethanolic extract containing ethosome formulation.	[131]
Hinokitiol (HKL)	The heartwood of certain trees belonging to the Cupressaceae family (cypress family).	Nanocapsule	Poly(ε-caprolactone), cetyltrimethylamonium chloride, and octyl salicylate	Emulsion–diffusion method	C57BL/6 mouse	The nanocapsules containing HKL are positively charged, providing electrostatic contact with the skin, which is likely promoting hair growth. The in vivo hair growth-promoting effects of the two preparations were comparable to those of the minoxidil 3% solution.	[132]
Poly (γ-Glutamic Acid)		Chitosan Hydrogel Nanoparticles	Acetic acid and chitosan	Ionic gelation	C57BL/6N female telogenic mice	Dermal papilla cells with poly (γ-glutamic acid) hair growth-promoting herbal extract mixtures showed an enlargement of hair bulbs and notable shape changes, respectively, indicating a potential for hair growth induction.	[133]
Luteolin	Leaves, flowers, fruits, vegetables (stalks/roots), and seeds/hulls	Nanoemulsions	Lipoid P75-3, poly (ethylene oxide)-block-poly(ε-caprolactone) (PEO-b-PCL)	Phase inversion composition	C57BL/6 male mice	The luteolin-loaded nanoemulsions exhibited hair growth-promoting activity, which is equivalent to using a luteolin solution in an organic solvent. Nanoemulsions were compared to minoxidil 3%.	[134]
Cedrol	Wood		Medium chain oil Span 80	_	C57BL/6 Mice	Cedrol nanoemulsion had positive effects on hair growth and enhanced drug solubility, compared with the ointment. 50 mg/mL cedrol nanoemulsion showed stronger hair growth than the 2% minoxidil and the ointment groups.	[135]
Cardamonin (CAR)	Several plant parts, primarily from plants belonging to the Ginger family (Zingiberaceae)	Liposomes	Phospholipid and cholesterol	Thin-film hydration	Rat	Loading CAR into liposomal gel improved the cumulative release of CAR. Cellular uptake of CAR-liposomes significantly increased compared with the drug-free solution. CAR-liposomes increased the topical content of the drug in the HF compared with free CAR.	[136]
Baicalin (BA)	Root and bark	Black phosphorus nanosheets encapsulated MN	NH2-PEG and black phosphorus	Liquid-phase exfoliation	SD rats	BP’s photothermal effects enhance BA delivery through the skin via MN. In vitro, efficacy is promoted by follicle cell proliferation and regulated gene expression related to hair growth. In vivo, efficacy led to faster hair regrowth in an AGA animal model, with less frequent dosing compared to commercial minoxidil tincture.	[137]

### 4.4. Solid Lipid Nanoparticles (SLNs)

Limitations observed from colloidal carriers, such as emulsions, liposomes, and polymeric nanoparticles, could be solved by incorporating drugs into SLNs, because they offer an optimum release profile and targeted drug delivery with a good physical stability [138]. The lipids used in preparing SLNs could be triglycerides, partial glycerides, fatty acids and steroids, and waxes, all stabilized by various emulsifiers and combinations [139]. The SLN formulations proved their effectiveness in the topical administration and a good localization of the drug in the skin to treat androgenic alopecia [140]. SLNs succeeded in highly encapsulating phytomedicines with a better permeability to the HF [141]. The Platycladus orientalis-loaded SLN was recommended for hair loss therapy [142].

### 4.5. Nanostructured Lipid Carriers (NLCs)

They are made up of an unstructured solid lipid matrix, including a blend of solid and liquid lipids and an aqueous phase with a surfactant or surfactant mixture. Solid lipids are typically combined with liquid lipids in a 70:30 or 99.9:0.1 ratio, with surfactant concentrations ranging from 1.5% to 5% (*w*/*v*) [143]. NLCs are divided into three categories based on their lipid content and formulation variables: imperfect, amorphous, and multiple-structure [144]. NLCs are drug delivery devices comprising solid and liquid lipids as the core matrix. It has been demonstrated that NLCs have some advantages over conventional carriers for drug therapy, including a higher solubility, the capacity to improve storage stability, an improved permeability and bioavailability, fewer side effects, a longer half-life, and a tissue-targeting delivery. NLCs have gained popularity in recent years [145]. NLCs possess the ability to penetrate the scalp [137]. The NLCs enhance Cinchonine activity, stimulating the hair growth and regeneration under AA conditions [127].

### 4.6. Transfersomes

Transferosomes are ultra-deformable vesicular carrier systems used for drug delivery, crossing skin barriers, and providing medication into deeper tissues due to their ability to squeeze through membranes [146]. Being so bendable enables a more efficient penetration of entire vesicles. As a result, it offers excellent potential for usage in innovative drug delivery systems [147]. Transfersomes are a type of vesicle-forming component made from an amphipathic ingredient, 10–25% surfactants/edge activators, and a hydrating medium. The main ingredient is amphipathic, while the surfactants and edge activators are biocompatible compounds. The solvent is typically ethanol or methanol [148]. Transfersomes containing nanosized particles have been proposed as promising drug delivery alternatives for treating localized hair problems, such as alopecia, compared to traditional dose forms, such as solutions [149].

### 4.7. Ethosomes

Classical ethosomes are modified forms of classical liposomes with a superior transdermal drug delivery because of their smaller size, negative ζ-potential, and higher entrapment efficiency. They are composed of phospholipids, a high concentration of ethanol (up to 45% *w*/*w*), and water [150]. Ethosomes have been shown to be successful in transporting compounds to and through the skin into the systemic circulation [151]. Ethosomes are made of components that are generally considered safe (GRAS) [152]. They act by increasing the cell membrane’s lipid fluidity due to the presence of ethanol in ethosomes, resulting in an increased skin permeability [153]. Moreover, choosing and concentrating on a suitable phospholipid is an important stage in determining the ethosomes’ effective penetration into the skin [154]. Ethosomal formulations could combine herbal extracts for the potential enhancement of the treatment of alopecia [131].

**Figure 3:**
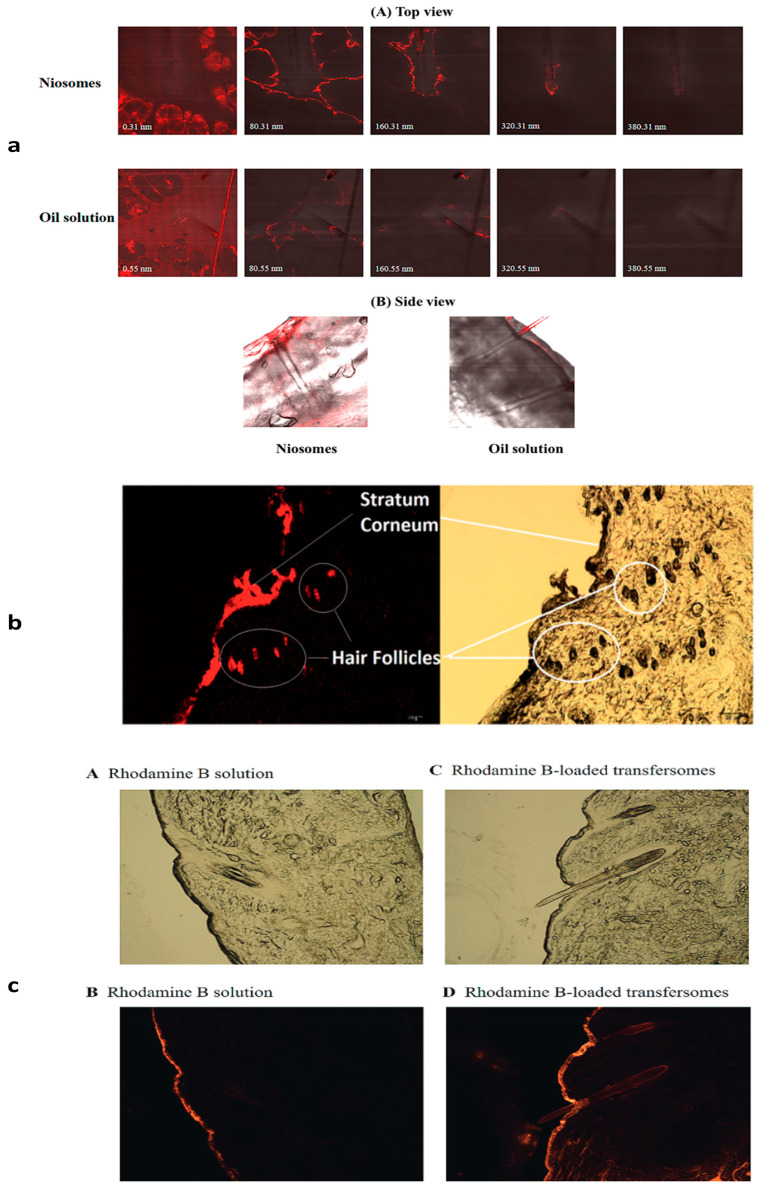
Confocal microscopic pictures display penetration of different nanosystems through HF using dye. (**a**) Penetration of nile red-loaded niosomes across pig ear skins in top view (**A**) and side view, (**B**) compared with control (nile red oil solution) [121]. (**b**) Penetration of Rhodamin B solution into hamster skin against application of 0.001% Rhodamin B-loaded SLN for 0.5 h [140]. (**c**) Subfigure (**A**,**C**) bright field images and (**B**,**D**) fluorescence images of the penetration of Rhodamin B solution into pig ear against Rhodamin B-loaded transferosmes for 30 min [149].

Owning to the tiny size and lipidic structure of lipid-based nanocarriers, they can penetrate and distribute easily through biological cells [155]. Figure 3 displays the penetration of different nanosystems containing dye through HFs against their dye solutions.

## 5. 3D Printing for Alopecia

### 5.1. History of Dermal 3D Bioprinting

Cell-based therapies, particularly stem cells, have a significant regenerative potential. However, a traditional two-dimensional (2D) cell culture often fails to replicate the 3D environment experienced by stem cells [156]. In recent years, engineering approaches have been developed to replicate this environment. 3D bioprinting has attracted considerable attention for its potential to replicate natural tissues, create a spatially organized environment for cells, and regulate the distribution of bioactive substances within scaffolds [157]. As a result, 3D bioprinting has been investigated for its application in developing biomimetic dermal–epidermal structures [158]. Table 2 represents a comparison between dermal 2D and 3D bioprinting.

**Table 2:** A comparison between 2D and 3D HF bioprinting.

Features	2D Bioprinting HF	3D Bioprinting HF	Refs.
Structure	Two-dimensional single-layer simple structure.	The 3D layer-by-layer complex structure resembles native HFs.	[159]
Cellular and biomaterials	Graphene oxide, cellulose, chitin, and proteins.	Dermal papilla cells, HUVECs, keratinocytes, and melanocytes in collagen–dermatan sulfate matrices, gelatin–alginate hydrogel.	[158,160,161]
Function	Supports basic cell studies.	Creates more realistic HF constructs that closely resemble the native HF structure.	[162]
Applications	Investigate signaling pathways of skin illnesses, such as psoriasis, or melanoma wound healing and test the efficacy of safe therapies.	Facilitates cell migration, mimicking a native-like microenvironment essential for angiogenesis, neurogenesis, proliferation, and differentiation.	[163,164]

### 5.2. Applications of 3D Printing for HF Generation

Although none of the current reconstructed skin models available contain fully developed HF units, Catarino et al. incorporated structures named spheroids created by printing dermal papilla cells and human umbilical vein cells (HUVECs) [165]. These spheroids were precisely printed within a pre-gelled dermal layer containing fibroblasts, and upon maturation, the resulting tissue developed HF–like structures [166]. In a study by Aliyazdi et al., living human HFs were transplanted into a collagen matrix within a 3D-printed polymer scaffold to replicate the follicle’s microenvironment [167]. This study presents an innovative approach using a 3D in vitro organ culture system with human HFs to investigate the hypothesis that antibiotic nanocarriers may reach bacteria within the follicular cleft more effectively than free drugs [167]. A 3D bioprinter was used to create hair-inductive tissue grafts, with collagen droplets containing mesenchymal and epithelial cells placed adjacent to each other for three days of culturing and then transplanted into the skin for hair growth [168].

A novel technique allows a controlled production of self-aggregating dermal papilla cells’ spheroids of a gelatin–alginate hydrogel in a physiologically realistic extracellular matrix, as well as the beginning of epidermal–mesenchymal interactions that lead to the formation of HFs in vivo [159]. Another in vivo study developed a bioprinting robot that used skin-derived precursors from newborn mice to achieve the in situ regeneration of HF-equipped skin [155,156]. Unlike traditional layer-by-layer deposition and subsequent transplantation, this novel method enables one-step printing onto skin defects [162]. Gelatin methacrylate and matrigel are effective substrates that aid in the survival and differentiation of these delicate progenitors [157,158]. Furthermore, HF-like extensions were formed by the intraoperative bioprinting of a human adipose-derived extracellular matrix and stem cells, indicating the role of adipocytes in matrix structuring and down-growth development [164].

A recent strategy using 3D-printed pills for alopecia. These pills are made of bioderived and biodegradable materials at a cheap manufacturing cost and have been demonstrated to be usable in the lab setting. A subsequent pilot investigation of this prototype will aid in the tailored medical and AA treatment of patients in clinical settings [169].

**Figure 4:**
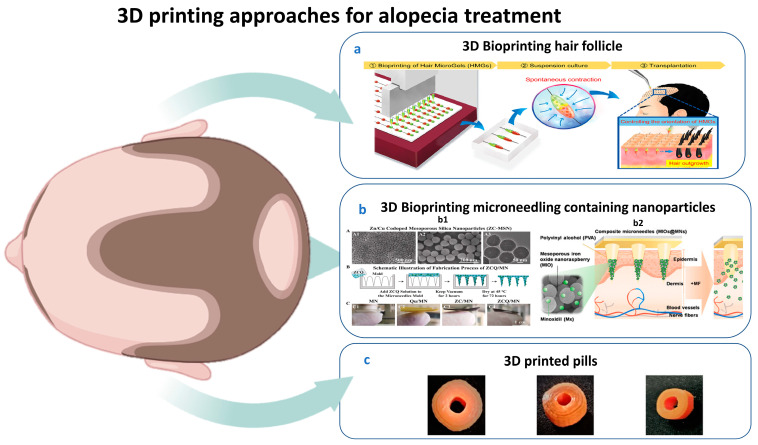
(**a**) 3D bioprinted hair microgel followed by spontaneous contraction and transplantation [168]. (**b**) 3D bioprinted microneedles containing (**c**) b1: fabrication process of zinc–quercetin microneedles, quercetin microneedles, zinc mesoporous silica nanoparticles microneedles, and quercetin-loaded zinc mesoporous silica nanoparticle microneedles [123]; b2: polyvinyl alcohol microneedles with dissolvable properties containing mesoporous iron oxide and nanoraspberry encapsulating minoxidil inside microneedles triggered by external magnetic field leading to increased temperature locally with controlled release [186]. (**c**) Baricitinib-incorporated PLA 3D-printed pills by fused deposition modeling [169].

For instance, in 2025 Wu et al. showed in an in vivo study the effect of calcium molybdate (CM) nanoparticle-based bioinks in supporting the long-term survival of dermal papilla cells and macrophages. In addition, multicellular scaffolds using CM nanoparticle-containing bioinks promoted hair regrowth by fostering an anti-inflammatory, immune microenvironment [170]. This approach can potentially be widely used in the field of HF engineering and related fields. Different 3D bioprinting techniques for alopecia are illustrated in Figure 4.

### 5.3. 3D Bioprinting Assisted by Artificial Intelligence and Machine Learning

Artificial intelligence (AI) and machine learning (ML) are now being used in the assessment of personalized scalp conditions [171]. Moreover, AI-related methods are utilized to predict hair loss patterns [172]. Furthermore, a recent study used various analysis methods of machine learning to identify the common hub genes as potential diagnostic marker genes linked to immunological responses and AA [173]. 3D bioprinting is regarded as a cutting-edge technology with numerous applications in healthcare, regenerative medicine, drug research, and tissue engineering, potentially offering eco-friendly opportunities [166,174]. For 3D-printed microneedles, ML models can estimate the needle quality even before design, resulting in cost- and time-efficient operations that make good use of materials and resources [175]. ML can identify design errors, optimize microstructures, reduce energy consumption, create substitutes, and predict drug release in 3D-printed drugs by analyzing geometric details and 3D printing process parameters [176]. AI is significant in the 3D printing of MN-based devices in predicting drug release patterns, quality control, and biomarker levels. Furthermore, the autonomous 3D printing of microneedles employs an integrated system of the Internet of Things and ML [177].

### 5.4. Limitations of 3D Bioprinting

The act of printing itself has the potential to harm or even destroy delicate cells, so a high cell viability during and right after printing is challenging [178]. Replicating the complex in vivo microenvironment and obtaining sufficient quantities of the right types of cells remains difficult [179]. Bioinks must be biocompatible and possess suitable rheological properties to maintain structural fidelity, while the available materials ideal for cell survival lack good printability or mechanical strength, and vice versa [180]. Compared to traditional 3D printing, the range of materials suitable for use as bioinks is still relatively limited [180]. Deep inside a bioprinted construct, cells cannot efficiently eliminate waste products through simple diffusion or obtain enough oxygen and nutrients, so necrosis, or cell death, may develop [181,182]. The process itself is relatively slow, which becomes problematic when scaling up to human-sized tissues or organs [183]. Scaling up the bioprinting process from the lab scale to producing clinically relevant quantities of tissue grafts or organs reliably and cost-effectively is challenging [184]. Incorporating functional nerve networks into bioprinted constructs is an often overlooked but critical challenge [185]. Bioprinters, bioreactors, bioinks, and growth factors are expensive, while bioprinting also requires sterile facilities and highly skilled personnel, making the development and potential application of bioprinting very costly [184].

## 6. Future Directions

No therapeutic treatment other than MN combined with phytomedicinal treatment-loaded nanoparticles produced a significant enhancement in hair regrowth and a lower dose frequency than traditional treatments [187]. Recently, a biodegradable MN has been developed to offer prolonged drug release with biosafety and patient compliance [133]. The exploitation of the synergistic effect of combining herbal drugs, like cedrol, with minoxidil-loaded nanoparticles reduces the skin irritation of minoxidil, promotes hair regeneration, and enhances the permeation through the scalp [188]. Phospholipid–polymer hybrid nanoparticles are considered a promising approach for delivering phytomedicines through scalp hair as they possess the cytoskeleton structure of polymers with sustained release properties and the biomimetic behavior of biological lipids [126]. The production of herbal-extract-loaded SLNs for topical gels could be a cost-effective and commercial alternative to conventional drug delivery systems [189]. However, all of the previous studies are promising for alopecia treatment; some studies lack long-term stability and efficacy data. The study by Zhang et al. [123] showed promising results for quercetin-loaded zinc mesoporous silica nanoparticles, but the short duration (14 days) limits our ability to assess long-term efficacy and safety.

Additionally, mice models may not fully represent human hair growth cycles. We recommend that further studies should consider more extended treatment periods and human clinical trials to validate these findings as a future direction. We reviewed 3D bioprinted microneedles containing mesoporous iron oxide nanoraspberries loaded with minoxidil [186]. These composite microneedles offer a novel and synergistic drug delivery system for transdermal applications with a broad clinical potential. Further research exploring the substitution of minoxidil with phytomedicinal extracts could provide a valuable strategy for reducing adverse effects and allergic reactions. While 3D bioprinting represents a novel strategy for HF regeneration, for circumventing the limitations associated with traditional transplantation, significant advancements are still required. Key areas of ongoing investigation include optimizing the longevity of bioprinted follicles and refining biomaterial compositions to more closely recapitulate the complex architecture and functionality of native HFs.

## 7. Conclusions

This review has focused on recent developments in delivering phytomedicines for alopecia treatment via different nanocarriers. Nano-drug delivery systems have garnered attention in various industries because of their outstanding physicochemical features. Hair-restoring treatments are on the rise nowadays. The current methods for hair restoration are harmful and produce unfavorable side effects. Numerous innovative nanocarriers are developed to enhance skin permeation and improve the sustained and controlled release without harnessing invasive methods. Moreover, the direction of using natural drugs provides a safer and more efficient treatment for alopecia. More studies are needed to examine the use of phytomedicine-loaded nanocarriers for greater drug targeting and fewer side effects. Nanocarriers, such as niosomes, liposomes, microemulsions, nanoemulsions, SLNs, ethosomes, and NLCs, have substituted the traditional drug delivery. Many herbal extracts and phytoconstituents are beneficial in alopecia treatment, offering a long-term biocompatibility with the skin. Since nanocarriers loading phytomedicines succeeded in in vivo animal models, it would be necessary to carefully test phytomedicinal nanopreparations on clinical patients. Therefore, in a nutshell, a collaboration of phytotherapy with nanoscience would be worthful in treating different scalp diseases.
